# Optical Determination of Lead Chrome Green in Green Tea by Fourier Transform Infrared (FT-IR) Transmission Spectroscopy

**DOI:** 10.1371/journal.pone.0169430

**Published:** 2017-01-09

**Authors:** Xiaoli Li, Kaiwen Xu, Yuying Zhang, Chanjun Sun, Yong He

**Affiliations:** College of Biosystems Engineering and Food Science, Zhejiang University, Hangzhou, China; Universidade de Lisboa Instituto Superior de Agronomia, PORTUGAL

## Abstract

The potential of Fourier transform infrared (FT-IR) transmission spectroscopy for determination of lead chrome green in green tea was investigated based on chemometric methods. Firstly, the qualitative analysis of lead chrome green in tea was performed based on partial least squares discriminant analysis (PLS-DA), and the correct rate of classification was 100%. And then, a hybrid method of interval partial least squares (iPLS) regression and successive projections algorithm (SPA) was proposed to select characteristic wavenumbers for the quantitative analysis of lead chrome green in green tea, and 19 wavenumbers were obtained finally. Among these wavenumbers, 1384 (C = C), 1456, 1438, 1419(C = N), and 1506 (CNH) cm^-1^ were the characteristic wavenumbers of lead chrome green. Then, these 19 wavenumbers were used to build determination models. The best model was achieved by least squares support vector machine (LS-SVM)algorithm with high coefficient of determination and low root-mean square error of prediction set (R^2^_p_ = 0.864 and RMSEP = 0.291). All these results indicated the feasibility of IR spectra for detecting lead chrome green in green tea.

## Introduction

Tea has been widely used as a flavored and healthy beverage in the world [1, 2]. Among all the organoleptic characteristics, color is regarded as an important quality indicator of tea [3]. Tea color is produced by pigments and their decomposed products, meanwhile, it reflects the retention of phenolic antioxidants. In order to make tea glossier, some tea producers illegally add lead chrome green into it, which may cause several adverse effects on human health [4]. Lead chrome green is a kind of industrial dye with a light green color, the main components of which are lead chrome yellow and phthalocyanine blue or prussian blue. These pigments are frequently used in painting and coating industries, with a large scale of production per year [5]. Nevertheless, lead exposure is well recognized for producing toxic effects in bones, gastrointestinal tract, kidneys, cardiac, reproductive and nervous systems [6]. Furthermore, adding any colorant in tea production is banned in China. So it is significant to detect the lead chrome green added illegally in tea, but there is still no standard method for detecting the lead chrome green in food.

At present, methods, which are used to analyze the existence of lead chrome green in tea, are simply based on the existence of lead or chromium [7, 8, 9, 10]. However, the accumulations of lead and chromium in tea may result from heavy metal pollution of soil and vehicle exhaust emissions in tea production process. Therefore, the existence of lead or chromium cannot confirm the existence of lead chrome green. Li et al. [11] used Raman spectroscopy to confirm the existence of lead chrome green in tea infusion. However, there are many difficulties of Raman quantitative detection, like the self-absorption of samples, the changes of refractive index caused by different concentrations of samples, the background noise from solvent and so on. In addition, the samples used in this reference [11] are tea infusion, but which in our study are tea powder. The tea powder is very difficult for Raman detection, because a strong fluorescence effect will be caused by the rich pigment compositions (chlorophyll, carotene) in tea powder. In addition, the traditional methods for detection of lead and chromium, based on chemical analysis, are chemical reagents-consuming, time-consuming and including a series of complicated procedures. Therefore, establishing a rapid, accurate and non-destructive detection method to evaluate the lead chrome green added in tea is necessary [3].

Infrared spectroscopy (IR) detects the functional groups in the molecules based on the changes of the vibrational energy level of molecules. More complex molecular structures lead to more absorption brands and more complex spectra. Especially, IR has been used for the characterization of very complex mixtures, and many components of complex mixtures can be simultaneously detected by a hybrid of the complex spectra and chemometrics. IR spectroscopy combined with chemometric algorithms has been widely applied to variety identification and quantitative detection in agriculture [12]. It has also been frequently used in the studies of tea analysis. Kokalj et al. [12] identified herbal tea by mid-infrared spectroscopy. Lee et al. [13] used the IR spectroscopy to determine the contents of caffeine and catechins in tea leaves. Li et al. [14, 15] used infrared spectroscopy to detect tea polyphenols content and dry matter content of tea. Recently, IR spectroscopy has been applied for pigment analysis. Chen et al. [16] characterized an eleven-layer automotive coating by Infrared spectroscopy. Miliani et al. [17] carried out a noninvasive study of ancient mural painting materials by using Fourier transform mid-infrared (mid-FT-IR) reflectance spectroscopy. However, there is little research on spectral determination of heavy metal dye in food.

IR spectrum includes a wide range of wavenumbers, which provide abundant information for modeling. However, these wavenumbers also have much redundancy and bring an extra computational burden. Thus, wavenumber selection is a very important step in dealing with spectra data. So far, many wavenumber selection methods have been utilized in spectral studies, including regression coefficient analysis (RCA) [18], successive projections algorithm (SPA) [19], interval partial least squares regression (iPLS) [20], and interval random frog (iRF) [21]. Among these methods, SPA and iPLS have been proved that they employ simple operation and demand a smaller computational workload. In addition, SPA and iPLS have steady performance and extensive adaptability, so they have been widely used in spectral studies. In this article, these two methods were chosen to select characteristic wavenumbers.

The aims of the paper are: (1) to analyze whether lead chrome green was added into the tea samples or not by IR spectra. (2) to determinate the concentration of lead chrome green in tea by IR spectra. (3) to build a simple and reliable model for measurement of lead chrome green in tea.

## Materials and Methods

### Sample preparation

Three brands of green tea samples were purchased at Zhejiang University Education Supermarket (120.2°E, 30.3°N, Hangzhou, Zhejiang province, China), and these brands included: Biluochun (BLC, originate from Suzhou, Jiangsu province, China), Maofeng (MF, originate from Huangshan, Anhui province, China) and Longjing (LJ, originate from Hangzhou, Zhejiang province, China). Lead chrome green was also purchased from three manufacturers included: Guang Zhou Hu An Pigment Company (LCG1, 113.3°E, 23.1°N, Guangzhou province, China), Shang Hai Ling Dong Chemical Company Lmt. (LCG2,121.3°E, 31.3°N, Hangzhou, Zhejiang province, China) and Xingtang Xinlei Mineral powder processing plant (LCG3, 114.5°E, 38.1°N, Shijiazhuang, Hebei province, China). Firstly, green tea of three brands was ground by a grinder mill (Tissuelyser-48, Shanghai, China) and the powders were sieved with a 60 mesh. Then the sieved LJ sample powder was divided into 135 parts, while the MF and BLC powder was divided into 30 parts respectively, each parts weighted 5.0 g. After which 0.0, 1.0, 2.0, 2.5, 3.0, 4.0, 5.0, 7.5, 10.0 and 12.5 mg lead chrome green of three different brands were mixed with 5.0 g sieved tea powder as shown in Table 1. To be more specific, lead chrome green powders of LCG1, LCG2 and LCG3 were added in to LJ tea powders, respectively. In the meantime, LCG1 was also added into BLC and MF tea powders, respectively. Successively, 1.0 g mixed powder was added into 49.0 g KBr medium, and mixed adequately for the following IR spectroscopy scanning. The total 195 sample were divided into a calibration set of 120 sample and a prediction set of 75 samples. More details were shown in Table 1.

**Table 1:** Statistical information of number of samples of each sample set.

Sample set	Calibration set					Prediction Set				
Concentration (mg/g)	0	0.4	0.6	1.0	2.0	0.2	0.5	0.8	1.5	2.5
LJ+LCG1	12	12	12	12	12	3	3	3	3	3
LJ+LCG2	3	3	3	3	3	3	3	3	3	3
LJ+LCG3	3	3	3	3	3	3	3	3	3	3
BLC+LCG1	3	3	3	3	3	3	3	3	3	3
MF+LCG1	3	3	3	3	3	3	3	3	3	3

### IR spectra acquisition

The IR spectra of samples were acquired by a Jasco FT-IR-4100 spectrometer (Tokyo, Japan) coupled with a TGS detector and a ZnO crystal sampling accessory in transmission mode. The detection range of spectrometer was 400–4000 cm^−1^ with resolution of 4 cm^-1^. And each spectrum was scanned 100 times, then the averaged spectrum was used for analysis. There was a high-strength ceramic light source inside and the 450 Michelson interferometer could be adjusted automatically. During the whole experiment, the temperature was kept at about 25°C.

Because of the system disturbance, there were obvious noises at the beginning and the end of the spectra. So, the first 400 and last 400 spectral data were deleted to avoid disturbance, and the following analysis were based on the spectra in range of 784–3581 cm^-1^.

### Elimination of outlier

**Fig 1:**
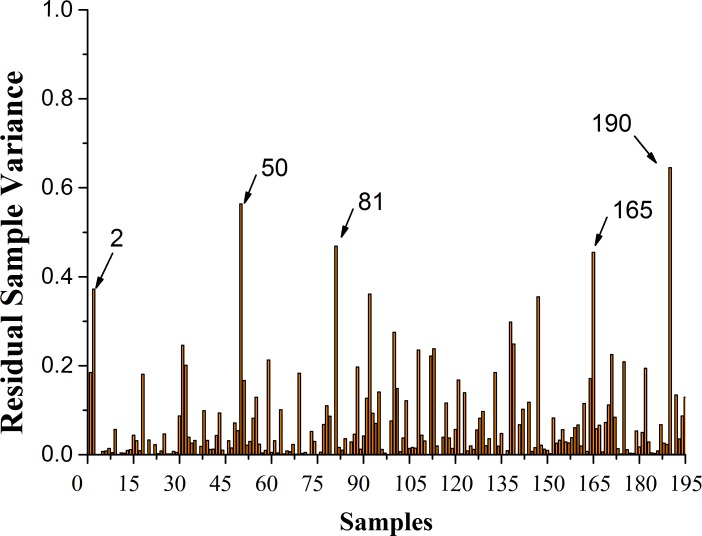
Residual sample variance of all samples.

The reliability of IR spectral analysis mainly depends on the accuracy and stability of the models. Abnormal sample is an important negative factor that affects the accuracy of model [22]. Therefore, elimination of abnormal sample is very useful to improve the model prediction capabilities. In this study, to judge whether a sample is abnormal or not depends on the variance of the residual samples, which was estimated by PLS, and the details were shown in Fig 1. Under normal circumstances, the greater the residual sample variance is, the weaker its ability to fit for correct model is, the less possible for it to explain [22]. It could be very clearly seen from Fig 1 that 5 samples, NO. 2, 50, 81, 165 and 190, had relatively high variance. Meanwhile, the amount of these 5 samples was only 2.56% of the whole samples. Therefore, these 5 samples could be regarded as the abnormal samples to be eliminated, and the remaining 190 samples were used for subsequent analysis.

### Data analysis

Partial least squares (PLS) regression is a most widely used statistical method for modeling independent variables (**X**) and dependent variables (**Y**) by linear multivariate relationship [23]. PLS is able to not only extract principal component from both input and output data, but also determine the direction on which input and output data have the largest covariance [24]. Partial least squares discriminant analysis (PLS-DA) is a PLS regression of a set **Y** of binary variables describing the categories on a set **X**. It is particularly suitable to deal with a much bigger number of **X** than observations, and with multicollinearity among **X**. In this research, PLS with cross-validation was used to find out whether the lead chrome green was added or not based on Matlab 7.0, and all the PLS models were established based on the full-cross validation.

IR spectral data contain hundreds of wavenumber variables. So, selection of optimal wavenumbers is significant for building a simplified model [20, 25]. Interval partial least squares (iPLS) regression is a wavenumber selection method proposed by Norgaard [20], which can extract the spectral wavenumbers highly related to the chemical structure, thus achieving the objective to improve the stability of the prediction model and increase the interpretability of the relationship between the spectral response and chemical structure [20].The successive projections algorithm (SPA)proposed by Araújo et al. [26] has also been proved to be a useful and effective tool for variable selection, which solves the collinearity problem with minimal redundancy [27]. In this study, a hybrid of iPLS and SPA were utilized to select the fingerprint wavenumbers of lead chrome green for exploring the quantitative relationship between IR spectra and the concentration of lead chrome green.

In order to obtain a better determination model, least squares support vector machine (LS-SVM) was used to build a nonlinear model and made a comparison with the linear model acquired by PLS. LS-SVM is an evolutionary version of the standard support vector machines (SVM) and has been introduced for the optimal control of nonlinear systems and spectral calibration [28].

In this paper, all the models including PLS-DA, iPLS, SPA and LS-SVM were performed on Matlab 7.0 (The Math Works, Natick, MA, USA). And the performance of models was evaluated by coefficient of determination and root-mean square error of calibration and prediction sets (R^2^_c_,R^2^_p_, RMSEC, RMSEP). If the value of coefficient of determination is more close to 1 and the value of root-mean square error is more close to 0, the model will have better performance.

## Results and Discussion

### Overview of samples' IR spectra

**Fig 2:**
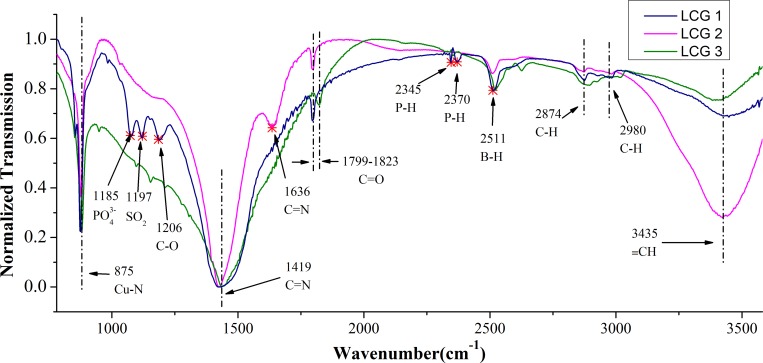
Normalized IR spectra and the characteristic wavenumbers of the samples of lead chrome green from three brands: LCG1, LCG2, and LCG3. For more details, see the text.

**Fig 3:**
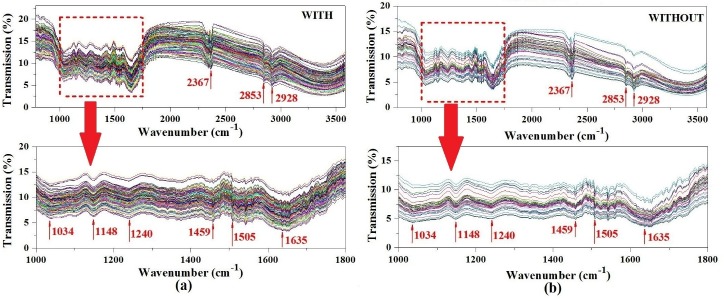
Original IR spectra of samples with lead chrome green (a) and samples without lead chrome green (b).

The IR spectra of three brands of lead chrome green were presented in Fig 2. It can be found that there are 7 main peaks in the all three spectra, including C-H stretching vibrations at 2874cm^-1^ and 2980 cm^-1^, C = N stretching at 1419 ~ 1435 cm^-1^ and 1636 cm^-1^ from aromatic groups, Cu-N stretching at 875 cm^-1^, C-O stretching vibration at 1206 cm^-1^, C = O at 1799 cm^-1^ and ≡CH at 3435 cm^-1^ [29, 30]. According to reference [30], the C = N vibration peaks found at 1419 ~ 1435 cm^-1^ was from aromatic groups of phthalocyanine. And the band at 875 cm was consistent with Cu-N stretching of phthalocyanine-Cu, which reflects that the main components of lead chrome green in our research were lead chrome yellow and phthalocyanine blue. Meanwhile, there are also some unique peaks among the three different brands of lead chrome green, which can be found at 1185, 1197, 2345, 2370 and 2511 cm^-1^, these different peaks indicated that there are different ingredients in the three brands of lead chrome green, which may caused by some impurities brought from different processing technology during the production of lead chrome green. Fig 3 showed the original spectra of a set of samples which are LJT powders, including those with LCG1 (**With**) and those without (**Without**). It can be seen that there was no obvious difference between the two groups of samples. Meanwhile, the main absorption peaks were observed in both Fig 3A and Fig 3B, including P-H stretching vibration of 2367 cm^-1^ and methylene C-H stretching vibrations of 2853 cm^-1^ and 2928 cm^-1^[29]. Especially, more absorption peaks were detected in the range of 1000–1700 cm^-1^, which were the fingerprints of these IR spectra. In this fingerprint region, some obvious absorption peaks were described as follows: sulfonic acids -SO_3_H (1034 cm^-1^), C-O stretching vibration (1240 cm^-1^), methyl C = N stretching (1459 cm^-1^), C = C stretching vibration (1505 cm^-1^), and C = N stretching vibration (1148 cm^-1^ and 1635 cm^-1^) [30,31, 32, 33]. In short, it is very difficult to figure out whether those tea samples were added lead chrome green or not with naked eyes based on the original IR spectra. Consequently, the chemometric methods were utilized for further analysis.

### Qualitative identification of lead chrome green added in tea

**Fig 4:**
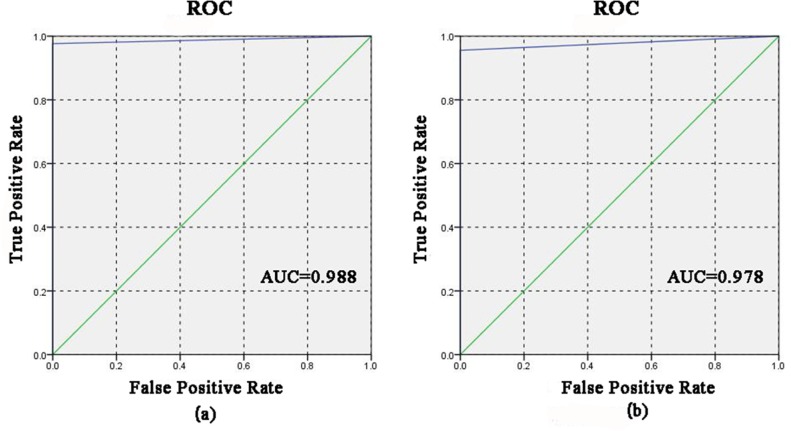
Receiver operating characteristic (ROC) curve of calibration set (a) and prediction set (b)

As seen in Fig 3, the spectra of all samples were quite similar and no distinct differences can be directly observed. It is very difficult to find out whether the tea samples were added lead chrome green or not with naked eyes. Consequently, qualitative analysis was carried on the IR data by PLS-DA. In the process of PLS-DA, samples without lead chrome green were assigned as 0, and samples added with lead chrome green were assigned as 1 manually. When the predicting value was bigger than -0.5 and smaller than 0.5, this sample was considered as **Without**. While the predicting value was bigger than 0.5 and smaller than 1.5, this sample was considered as **With**. The correct rate of **Without** and **With** for both calibration and prediction sets were all 100%, which means the tea samples had been clearly classified. To show the performance of PLS-DA more clearly, receiver operating characteristic (ROC) curve was used in this study, shown in Fig 4. ROC curve is a common method to show the efficiency of classification. It can be seen from Fig 4A and Fig 4B, ROC curve of both calibration and prediction sets were above the diagonal. And the area under curve (AUC) of calibration and prediction sets were 0.988 and 0.978, respectively, which reveals a good classification result. In a word, all the results showed that IR spectroscopy had the potential to distinguish the tea samples with lead chrome green from those without.

### Quantitative detection of lead chrome green added in tea

#### Establishment of linear model

**Fig 5:**
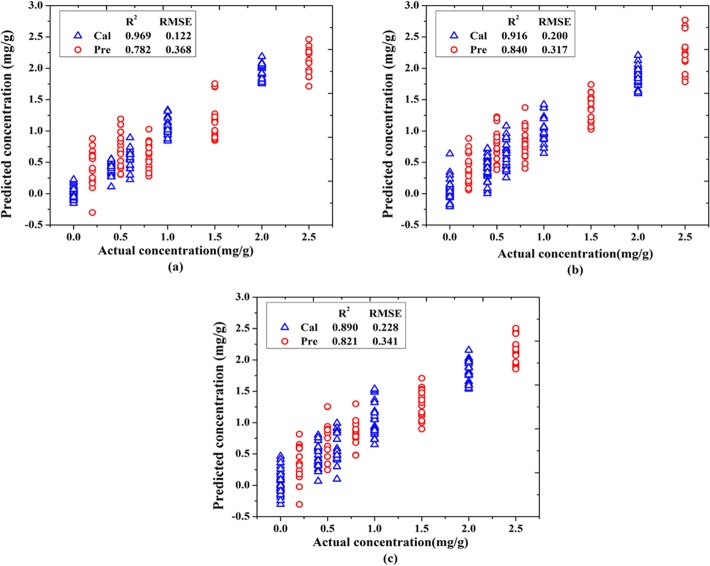
Scatter plots of actual vs. predicted concentration of Model 1 (a), Model 2 (b) and Model 3 (c).

On account of the difficulties of quantitative detection by chemical analysis methods mentioned in the introduction, IR spectroscopy coupled with chemometric methods was used for quantitative detection of lead chrome green in tea. Before analysis, in order to extract the optimal characteristic wavenumbers for this determination, a full spectrum (784–3581 cm^−1^) determination model (Model 1) was firstly built as a reference standard for evaluation. The results of Model 1 were shown in Fig 5A, values of R^2^_c_ and R^2^_p_ were 0.969 and 0.781, respectively, and values of RMSEC and RMSEP were 0.122 and 0.368, respectively. So it can be concluded that the accuracy of Model 1 was good. However, the wavenumbers utilized in Model 1 were too many, which resulted in a long modeling time and a complex structure between X and Y variables. In order to reduce the model operation time and increase the model accuracy, the characteristic wavenumbers need to be extracted to improve the model.

**Fig 6:**
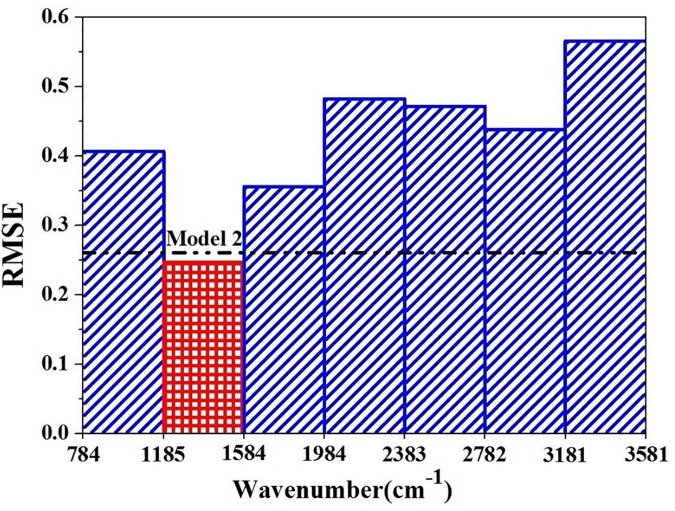
Extraction of characteristic wavenumbers by iPLS.

In this study, iPLS and SPA methods were hybridized to extract the characteristic wavenumbers for determination. The process of iPLS consists of splitting the spectra into equal-width intervals and developing sub-PLS models for each one, and then the sub-interval with the lowest value of RMSE are chosen as the best one [34]. In this study, the full spectrum from 784 to 3581 cm^−1^ was firstly equally split into 7 subintervals and each subinterval was used to build a PLS model. As shown in Fig 6, the abscissa represents the wavenumbers and the ordinate represents the RMSE of each model. The RMSE value of the global model with the full spectrum was represented by the dotted line. It can be found that Model 2 (1185–1584 cm^-1^) has a lower RMSE value than that of the global model. Details of Model 2 were shown in Fig 5B. It can be found that values of R^2^_c_ and R^2^_p_ were 0.916 and 0.840, respectively, and values of RMSEC and RMSEP were 0.200 and 0.317, respectively. Compared with Model 1, Model 2was more stable. As the values of R^2^_c_, R^2^_p_, RMSEC and RMSEP were all much better, and the gap between calibration and prediction was more smaller. All the above results indicated that the wavenumbers of 1185–1584 cm^-1^were the spectral characteristic range of lead chrome green.

**Fig 7:**
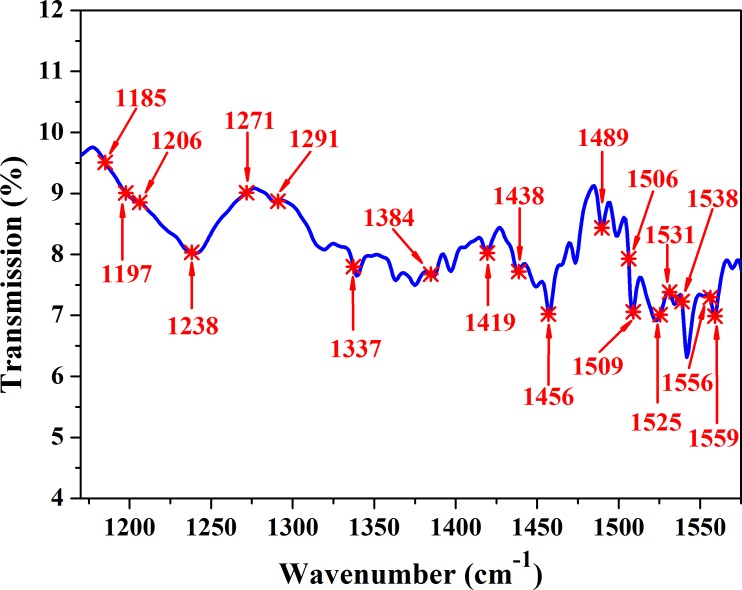
IR spectrum and the 19 characteristic wavenumbers of lead chrome green selected by SPA.

After selecting the wavenumbers range based on iPLS, the accuracy of the PLS model has been greatly improved, and the wavenumbers used during the modeling were also reduced from 2397 to 400. However, this range selected by iPLS still has some redundant and uninformative variables. In order to achieve the goal that using the least wavenumbers to establish the best model, SPA algorithm was used to extract characteristic wavenumbers in the range of 1185–1584 cm^-1^. In the process of SPA, the amount of characteristic wavenumbers was limited between 5 and 30. Then, 19 characteristic wavenumbers were selected, as shown in Fig 7. And the detail attributions of these 19 wavenumbers were listed in Table 2. Combination of Fig 2 and the other references[29,30, 31, 33, 35, 36], including 1384 (C = C), 1456, 1438, 1419 (C = N), and 1506 (CNH) cm^-1^ were the characteristic wavenumbers of pyrrole from phthalocyanine blue, which reflects that the main components of lead chrome green were lead chrome yellow and phthalocyanine blue. Subsequently, these 19 wavenumbers were used to build a PLS model (Model 3), and the result of Model 3 was shown in Fig 5C. It can be seen that values of R^2^_c_ and R^2^_p_ were 0.891 and 0.820, respectively, and values of RMSEC and RMSEP were 0.228 and 0.341, respectively. All the results of different wavenumber selection methods were shown in Table 3. Compared with Model 2, the performance of model 3 was slightly worse, but was still impressive, as the dimension of wavenumbers (independent variable) in Model 3 is only 4.75% of that in Model 2. While, Model 3was comparable with Model 1. In detail, the R^2^_c_, R^2^_p_, RMSEC and RMSEP of Model 3 were quite close with those of Model 1. Furthermore, there was a smaller difference between R^2^_c_ and R^2^_p_ (or RMSEC and RMSEP) of Model 3 than that of Model 1. More than that, the most obvious advantage of Model 3 was that the modeling wavenumbers (independent variable) were only 19, which was only 0.16% of the wavenumbers used in Model 1. That is to say, Model 3 achieved the goal by using the least wavenumbers to establish a wonderful model. And it could be concluded that these 19 wavenumbers were the most powerful feature for representing the spectral characteristic of both additive (lead chrome green) and tea.

**Table 2:** Attribution of main characteristic wavenumbers.

Characteristic wavenumbers (cm^-1^)	Functional group/Chemical bond
1185	Phosphate out-of-phase stretch (PO_4_^3-^)
1197, 1238, 1291	Sulfones (SO_2_)
1206	C-O stretch of carboxylic acids
1384	Pyrrole C = C stretch
1419,1438, 1456	C = N stretch of aromatic groups (C = N)
1489, 1525, 1531, 1538, 1556, 1559	Aliphatic nitrates (N = O)
1506, 1509	CNH group of aromatic groups

**Table 3:** Results of different wavenumber selection methods.

Model No.	Methods	Selected wavenumbers (cm^-1^)	Variable number	R^2^_c_	RMSEC	R^2^_p_	RMSEP
Model 1	None	784–3581	2397	0.969	0.122	0.782	0.368
Model 2	iPLS	1185–1584	400	0.916	0.200	0.840	0.317
Model 3	iPLS+SPA	1185,1197,1206,1238,	19	0.891	0.228	0.821	0.341
		1271,1291,1337,1384,					
		1419,1438,1456,1489,					
		1506,1509,1525,1531,					
		1538,1556,1559					

#### Establishment of nonlinear model

**Fig 8:**
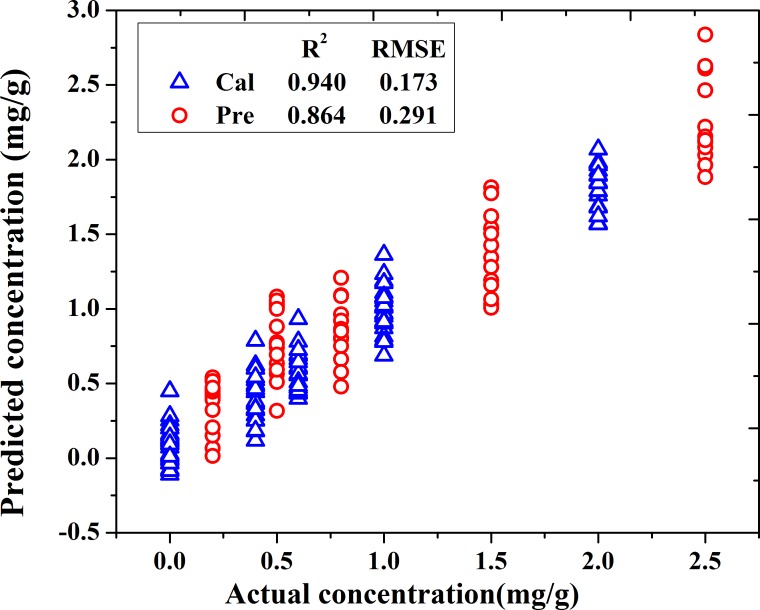
Scatter plots of actual vs. predicted concentration of LS-SVM model.

To further improve the accuracy of the model, LS-SVM was used to build a nonlinear model. In the process of LS-SVM, the wavenumbers used to build the nonlinear model were the 19 wavenumbers selected by a hybrid of iPLS and SPA, including 1185,1197,1206,1238,1271,1291,1337,1384,1419,1438,1456,1489,1506,1509,1525,1531,1538,1556 and 1559 cm^-1^. Before LS-SVM modeling, two main parameters (γ and δ^2^) should be first determined. After many trials and errors, the ranges of γ and δ^2^ were set as 10000–500000 and 100–30000, respectively. During the process, the optimal values of γ and δ^2^ were obtained with *γ* = 6.041×10^4^ and δ^2^ = 2.299×10^3^. Then, the results of LS-SVM model were shown in Fig 8. It can be found that values of R^2^_C_ and R^2^_p_ were 0.940 and 0.864, respectively, and values of RMSEC and RMSEP were 0.172 and 0.291, respectively. Compared with the results of Model 3, this model had a great improvement in the accuracy. It satisfied the demand of reducing the model operation time and improving the accuracy of the model at the same time, so it was the best model for the determination of lead chrome green in tea.

## Conclusions

This research explored the feasibility of IR spectroscopy in the determination of lead chrome green added in tea. Firstly, IR spectra were used in the qualitative analysis of lead chrome green addition, and the correct rate of With and Without for both calibration and prediction sets all reached 100%. Then, characteristic IR wavenumbers were selected by a hybrid of iPLS and SPA for quantitative detection of lead chrome green. 19 wavenumbers were selected as the key fingerprint of lead chrome green, and the nonlinear LS-SVM model based on these 19 wavenumbers achieved the optimal results with R^2^_p_ and RMSEP of 0.864 and 0.291, respectively. In a word, all these results proved that the IR spectroscopy has the potential to provide a fast, accurate and nondestructive analysis for the detection of lead chrome green in green tea.

## Supporting Information

S1 Dataset Calibration_Data. The original data of calibration set.
(XLSX)
